# Oligo(amylene) from the reaction of fusel oil with zinc dihalide†

**DOI:** 10.1039/d0ra10386a

**Published:** 2021-01-07

**Authors:** Matthew C. Davis, Laszlo Prokai, Josanne Dee Woodroffe

**Affiliations:** Naval Air Warfare Center, Michelson Laboratory, Chemistry Division China Lake California 93555 USA; University of North Texas Health Sciences Center, Department of Pharmacology and Neuroscience, Mass Spectrometry and Proteomics Laboratory 3500 Camp Bowie Boulevard Fort Worth Texas 76107 USA matthew.davis@navy.mil

## Abstract

Heating mixtures of fusel oil and zinc chloride or zinc bromide to 180 °C gave water, difusel ethers and the hydrocarbon oligo(amylene) as the major coproducts. Separation by chromatography gave oligo(amylene) in 25% yield from fusel oil. The triamylene fraction of the oligo(amylene) had a net heating value of 43.9 kJ g^−1^ which was 3% greater than specifications for gasoline, diesel #2 and jet A-1. The cetane number of the triamylene was 31.9 so it may not be useful for diesel engines. The triamylene had a flashpoint of 38 °C, viscosity (−20 °C) of 7.85 mm^2^ s^−1^, density (15 °C) of 0.78 g mL^−1^ and melting point below −78 °C which all compared well to the specifications of jet A-1.

## Abstract 2

Fusel oil was converted into the liquid hydrocarbon oligo(amylene) by zinc bromide catalysis.

## Graphical abstract

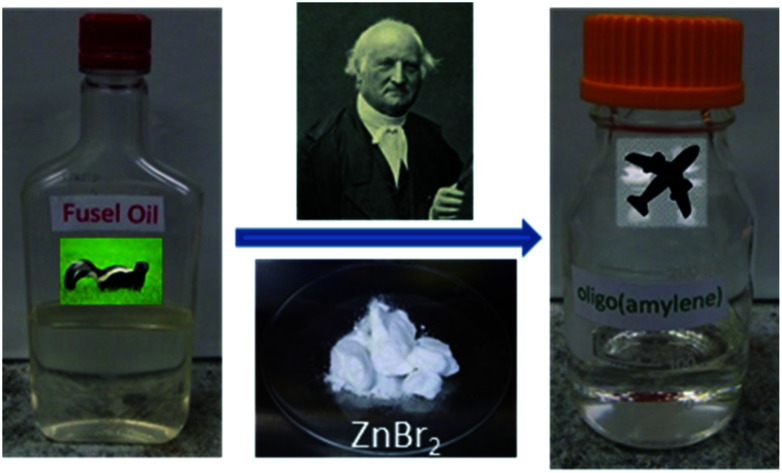

## 1. Introduction

**Fig. 1:**
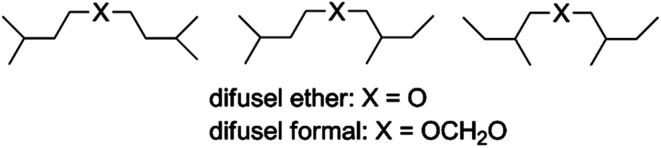
Structures of biodiesel oxygenated fuels synthesized from fusel oil.^7^

To maintain operational effectiveness, the armed services consume an enormous quantity of energy including liquid hydrocarbon fuels for air, land and sea vehicles.^1^ These liquid fuels are almost entirely derived from petroleum deposits found beneath the Earth's surface.^2^ The resulting crude oil from various parts of the world is processed, cracked, distilled and separated or ‘cut’ into the three most important fuel categories used by the military: aviation (jet), diesel, and gasoline.^3^ The US Navy has set ambitious goals of obtaining half of its energy from non-petroleum sources by 2020.^4^ We recently showed that fusel oil, pentanol isomers (isoamyl and 2-methyl-1-butyl alcohols) that are by-products from ethanol production, could be converted into ethers and acetals that combust properly in diesel engines (cetane numbers >40), Fig. 1.^5–7^ Although the energy density of the latter oxygenated fuels were lower than diesel #2, such biodiesel could be a useful blendstock for non-tactical transportation purposes.

For military jet fuels such as JP-8 (≥42.8 MJ kg^−1^), a decrease in energy density is not acceptable so oxygenated fuels are generally unsuitable for military aviation.^8,9^ Attempting to prepare jet fuel from fusel oil would require different chemical reactions yielding hydrocarbons devoid of oxygen. There has been some recent technology referred to as alcohol-to-jet (ATJ) which has been developed by several research groups.^10–14^ In these two-step processes, fermentation alcohols (ethanol, 1-butanol, isobutanol) are converted by high temperature (>300 °C) dehydration over catalyst (silica/alumina) to alkene. In the second step, the alkenes are oligomerized over special catalysts (noble metals; Ziegler or acidic zeolites and resins) into ATJ synthetic paraffinic kerosene (ATJSPK), which are hydrocarbons ranging from 8–16 carbons with suitable properties for aircraft gas turbine engines.^15^

**Fig. 2:**
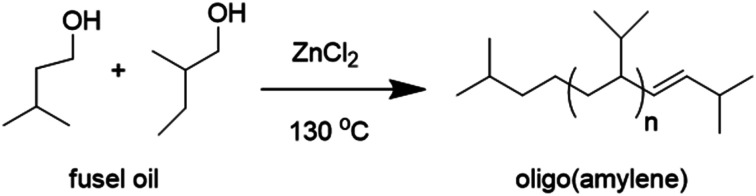
The oligomerization of fusel oil with zinc chloride reported by Balard.

While these contemporary technologies would likely be useful to convert fusel oil to hydrocarbon liquids, there were early chemical studies in the characterization of fusel oil which appeared interesting in this respect. In 1844, Antoine Jérôme Balard described the preparation of ‘amylene’ and its oligomers (oligo(amylene), *n* = 0–2) by heating a mixture of amyl alcohol from fusel oil and zinc chloride (ZnCl_2_), Fig. 2.^16–20^

Although Balard had combustion analyses that supported the identity of the products, he did not report any details regarding the chemical apparatus, reagent stoichiometry, conversion or percent yield of the products and did not have the benefit of modern chemical spectroscopy techniques. The reaction was mentioned in 1918 by Adams and coworkers who obtained little of the desired ‘amylene’ but a preponderance presumably of oligo(amylene).^21^ Although a complex product mixture was anticipated, we decided to reinvestigate Balard's reaction of fusel oil and zinc dihalides and this brief report will describe our preliminary results in isolating and characterizing the hydrocarbon products generated.

## 2. Experimental

### Materials and methods

**CAUTION!** The zinc chloride and zinc bromide salts are both toxic to humans when ingested.^22,23^ Operators should use all appropriate personal protective equipment (gloves, face shield, laboratory apron, fume cupboard, *etc.*) when handling these substances. Crude fusel oil was obtained from the Archer Daniels Midland Company and contained ∼13 wt% water and 7 vol% ethanol. Anhydrous zinc bromide and anhydrous zinc chloride were obtained from TCI America. Column chromatography was performed on silica gel (60 Å, 75–200 μm). Analytical thin-layer chromatography (TLC) was performed on Merck silica gel 60 F_254_ aluminum-backed plates. Visualization of TLC was by a UV light, 10 wt% phosphomolybdic acid (H_3_PMo_12_O_40_) in EtOH (PMA stain) and 0.75 wt% potassium permanganate (KMnO_4_) in H_2_O (KMnO_4_ stain). Details of additional methods to analyze the properties of the oligo(amylene) product including gas chromatography-mass spectrometry (GC-MS), bomb calorimetry, ignition quality test (IQT) and derived cetane number (DCN), elemental microanalysis, freezing point, flash point, density and kinematic viscosity and nuclear magnetic resonance (NMR) spectroscopy can be found in the ESI.†

### Oligo(amylene) from crude fusel oil

A borosilicate-glass, round-bottomed flask (1 L) equipped with magnetic stirring bar was filled with crude fusel oil (287.5 g, 2.84 mol) followed by anhydrous zinc bromide (320 g, 1.42 mol, 0.5 equiv.). A Dean–Stark trap (100 mL) was equipped to the flask along with a high-efficiency condenser cooled by a recirculating coolant chiller (6 °C). The reaction was heated with a heating mantle (Variac® 55%). The solids completely dissolved after a short time and the mixture was vigorously stirred. Once the internal temperature was sufficiently high (∼180 °C), a vigorous reflux was established. After the distillation/collection of H_2_O appeared to cease (85 mL; 7 h), the mixture was cooled to RT. The Dean–Stark trap was replaced with a distillation head and the reaction mixture was distilled at reduced pressure (10 Torr, Variac® 40%) to obtain an unfractionated distillate (80–120 °C, 134.89 g). The distillation pot contained the residual zinc bromide salt that was a slightly pinkish colored solid. The distillate was chromatographed through a short plug of silica gel eluting with hexanes to remove polar components and purify the hydrocarbon product (TLC: *R*_f_ = 0.9 (hexanes); PMA or KMnO_4_ stain). The collected fractions containing hydrocarbon product were rotary evaporated (60 Torr, 40 °C bath) to remove the hexanes solvent. Then the residue was distilled at reduced pressure (10 Torr, Variac® 40%) to obtain the final product as a clear, colorless mobile liquid (49 g, 24%).

## 3. Results and discussion

### Synthesis and characterization of Oligo(amylene)

**Fig. 3:**
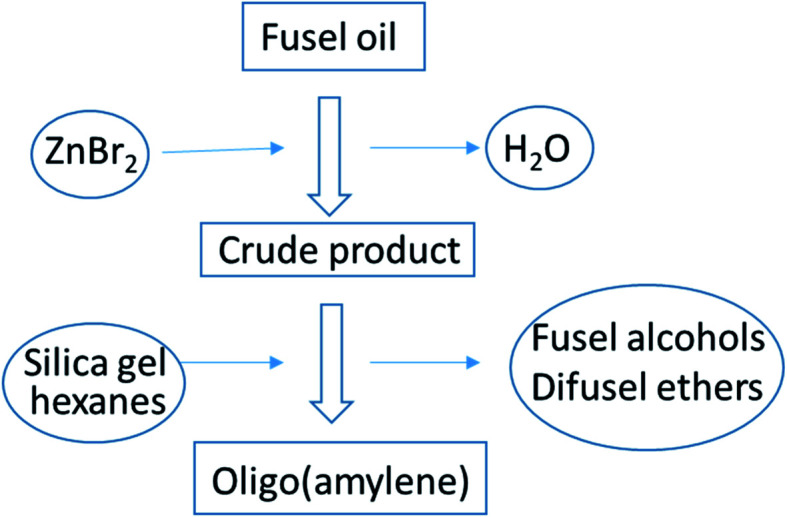
Process flow diagram for the preparation of oligo(amylene) from fusel oil by zinc bromide catalysis.

A ratio of crude fusel oil/zinc dihalide (2/1) was chosen as a starting point based on the work of Kuchkarev and Shuikin who prepared complexes between simple alcohols and zinc dihalides with this ratio.^24^ Heating these mixtures up to ∼180 °C brought about azeotropic removal of water indicating a dehydrative reaction had occurred. The amount of water collected was roughly equivalent to sum from the ‘wet’ fusel oil and the stoichiometric dehydration of fusel alcohols. Afterwards, a liquid crude product mixture was distilled away from the zinc dihalide salts. Analysis by thin-layer chromatography (TLC) showed the crude product was composed primarily of a nonpolar component moving at the solvent front along with a polar component near the origin. There was little difference in product mixtures between zinc chloride or zinc bromide reactions by TLC analysis. However, product mixtures were cleanly separable by distillation from zinc bromide (mp 394 °C), whereas zinc chloride (mp 275 °C) tended to ‘bump’ into the condenser during the later stages of distillation owing to its lower melting temperature.^25^ For these reasons it was decided to focus on the reaction between zinc bromide and fusel oil which may not have been reported previously.^26^ A general diagram of the process flow for the dehydration/oligomerization of fusel oil by zinc bromide and isolation of oligo(amylene) is shown and described below, Fig. 3.

**Fig. 4:**
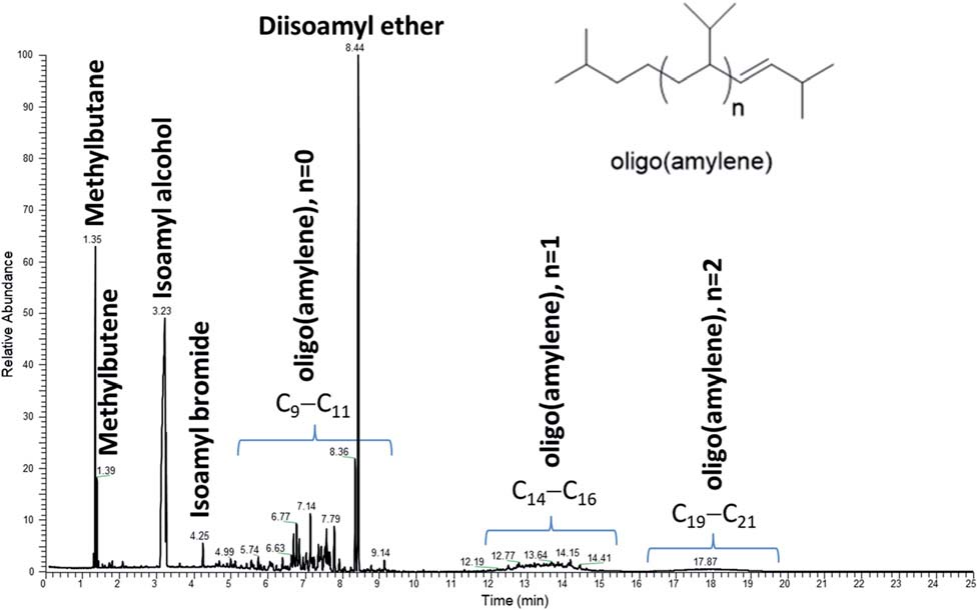
GC-MS chromatogram (total ion current, 70 eV) of the crude product from the reaction of fusel oil and zinc bromide.

More thorough investigation of the crude product by gas chromatography-mass spectrometry (GC-MS) showed major peaks for isopentane (1.35 min; 2-methylbutane), unreacted isoamyl alcohol (3.23 min) and diisoamyl ether (8.44 min), Fig. 4. Although anticipated to be a major reaction pathway, halogenation of isoamyl alcohol, the primary component of fusel oil, was very minimal since only a trace of isoamyl bromide (4.25 min) had formed. The only additional components of the reaction product were oligo(amylene) (*n* = 0, diamylene; *n* = 1, triamylene; *n* = 2, tetraamylene, *etc.*) that showed significant disproportionation and/or cracking (5–20 min).^27,28^ For example, the isopentene isomer (1.39 min) was accompanied by a significant peak of isopentane peak (1.35 min). It was expected that isoamyl alcohol would undergo dehydration from the zinc bromide catalyst leading to isopentene (3-methyl-1-butene). Furthermore, one would anticipate 3-methyl-1-butene to isomerize to the more thermodynamically stable 2-methyl-2-butene under the vigorous conditions of the reaction.^29^ However, it is difficult to conclude whether peak 1.39 min is either methylbutene isomer since their mass spectral fragmentation patterns are virtually identical.^30^ The oligo(amylene) tended to be mixtures complicated by elimination or addition of one carbon fragment. Thus, the diamylene had C_9_ and C_11_ portions, the triamylene had C_14_ and C_16_ and so on up to octaamylene (+C_29_ and C_31_) which appeared to be the heaviest oligomer formed under the reaction conditions. The oligomers were composed of many peaks most likely owing to isomers.

**Fig. 5:**
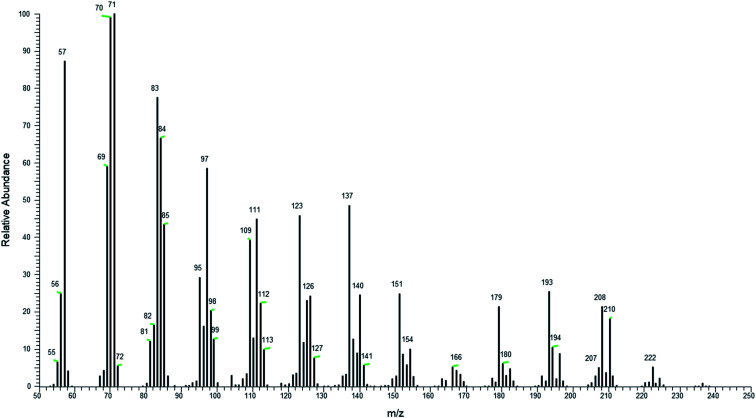
Averaged electron ionization mass spectrum (12 eV) of the triamylene domain (C_15_H_30_).

Of the oligo(amylene), the diamylene fraction was in greatest concentration, followed by triamylene and the heavier *n* = 2–4 were in lowest concentration based on the GC-MS total ion chromatogram. The mass spectrum of triamylene is illustrative of the fragmentation pattern of these hydrocarbons, Fig. 5. The base peak at *m*/*z* 71 (C_5_H_11_^+^) indicated a hydrocarbon as many small branched hydrocarbons share this base peak.^31^ The fragments are also separated by *m*/*z* 14 mass units which is the characteristic carbenium ion fragmentation found in hydrocarbons.^32,33^ Owing to the high degree of branching likely present in the oligo(amylene), the spectrum does not have the smooth exponential decrease in fragment peak height typical of linear hydrocarbons. Although the M^+^˙ at 210 is present which fits well with triamylene (*n* = 1), there were *m*/*z* 224 ion (*n* = 1 + CH_2_) and 196 (*n* = 1-CH_2_) from cracking processes.

**Fig. 6:**
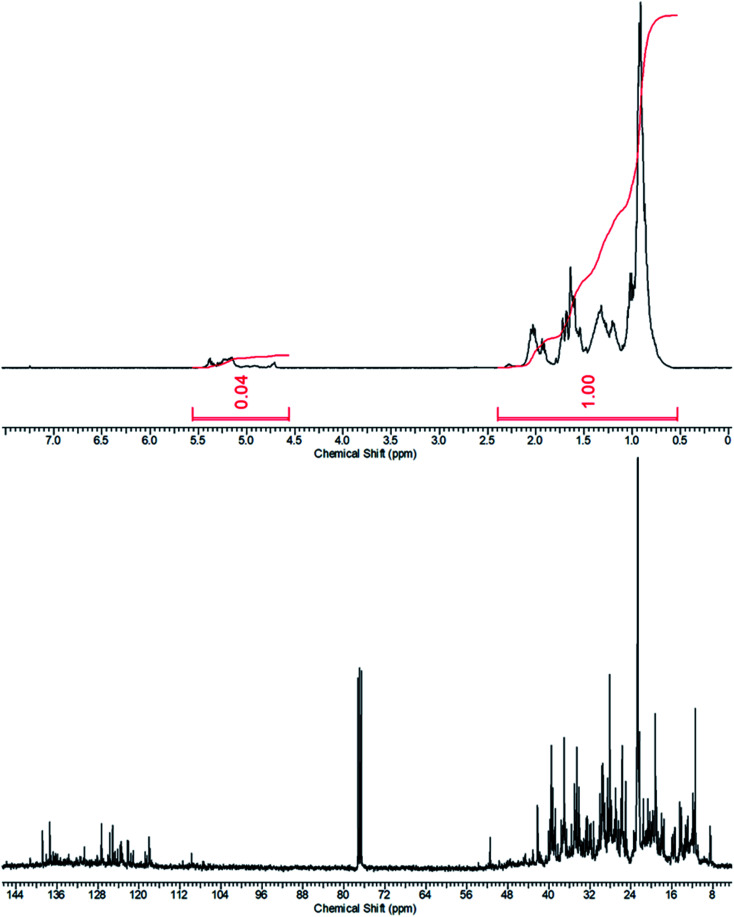
Proton and carbon–13 NMR spectra of the purified oligo(amylene) product in deuterochloroform at room temperature.

Attempts to separate the polar and non-polar components of the crude product mixture with the process equipment on hand failed as the two components appeared to co-distill at atmospheric or reduced pressure. Similar difficulties were experience by others in the fractional distillation of related mixtures of methyl *tert*-butyl ether and isobutene dimers.^34,35^ Therefore, the crude product mixture was fractionated by liquid chromatography on silica gel. The fast moving, non-polar component was cleanly separated by this process and the proton and carbon-13 nuclear magnetic resonance spectrum showed that it was the oligo(amylene), Fig. 6. The spectrum was free from signals related to fusel alcohols and difusel ethers (∼3.5–3.0 ppm).^7^ The spectrum was very simple with a less intense alkene region at 5.5–4.75 ppm and a dominant aliphatic hydrocarbon region 2.0–0.75 ppm with an integral ratio of 0.04 consistent with a product having one double bond. The carbon-13 spectrum was also relatively simple with an alkene region 140–115 ppm and an aliphatic region 50–8 ppm. The relatively small integrative value of the alkene region in the proton spectrum and the relatively large area of the alkene region in the carbon-13 spectra indicated that the alkenes of the oligo(amylene) were highly substituted (*e.g.* tertiary).

**Fig. 7:**
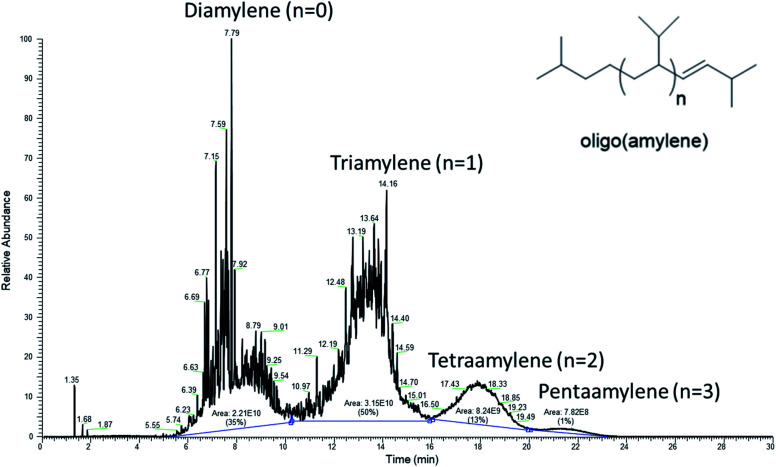
GC-MS chromatogram (total ion current, 70 eV) of the purified oligo(amylene) product.

Elemental composition (C and H) of the oligo(amylene) by elemental microanalyses were both within the commonly accepted margin of error (<0.4%) for the structure as drawn, Table 1. The microanalyses also proved that the hydrocarbon was free of bromine and verified the GC-MS data which lacked signature molecular ions for alkyl bromides and/or alkyl polybromides and their bromine isotopes.^36^ As further proof that the solvent from chromatography had been completely removed, GC-MS analysis corroborated the absence of hexanes in the post-chromatographic, oligo(amylene) product, Fig. 7. As anticipated, the volatile methylbutane and methylbutene were lost but lighter diamylenes were not removed during the hexanes evaporation. An estimate of the oligo(amylene) distribution based on the GC-MS total ion chromatogram was: *n* = 0 (35%), *n* = 1 (50%), *n* = 2 (13%), and *n* = 3 (1%). Interestingly, this product profile matched the original results of Balard who isolated only oligo(amylene) (*n* = 0–2).^16^ The yield of oligo(amylene) produced based on fusel oil input was calculated at ∼25% which was typical after a series of runs using either zinc chloride or zinc bromide. This yield did not take into account the mass of unreacted isoamyl alcohol nor the by-product diisoamyl ether which were not isolated during this brief study. Although a cold condenser was used during the reaction, it was likely that some 2-methylbutene isomers may have been lost to evaporation from the reactor system. In runs that were conducted for longer periods of time in the effort to achieve a higher yield of oligo(amylene), the yield did not increase but the oligomer distribution was shifted to higher number repeat units.

**Table 1:** Carbon and hydrogen microanalyses results for the purified oligo(amylene)

Molecular formula	Carbon (%)			Hydrogen (%)		
	Theory	Found	Difference	Theory	Found	Difference
(C_5_H_10_)_*n*_	85.63	85.53	0.1	14.37	14.19	0.18

**Fig. 8:**
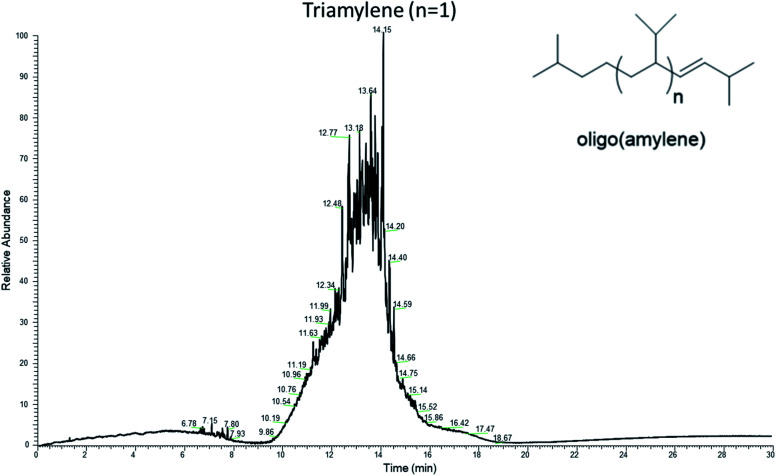
GC-MS chromatogram (total ion current, 70 eV) of the triamylene which was purified from the oligo(amylene) mixture by single fractional distillation.

It was fortuitous that the reaction conditions gave oligo(amylene) containing a preponderance of triamylene since it was hypothesized that this fraction would have fuel properties closest to kerosene type fuels (*e.g.* diesel and jet). The purified oligo(amylene) was fractionated by one distillation at reduced pressure which gave a single fraction that was primarily triamylene (oligo(amylene) *n* = 1) by GC-MS analysis, Fig. 8. The lighter diamylene fraction was lost during the reduced pressure distillation while the distillation pot residue was composed of the less volatile oligo(amylene) (*n* = 2–4). In one run where the reaction mixture was allowed to continue for a longer period of time, bulk quantities of the higher boiling tetraamylene were isolated by fractional distillation (ESI†).

**Fig. 9:**
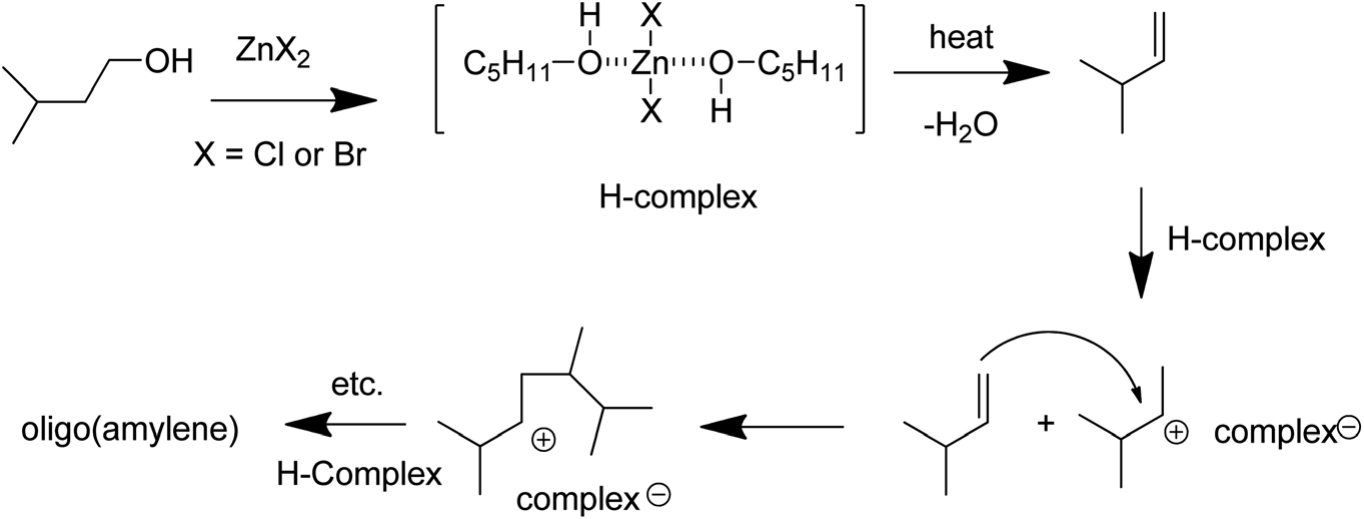
Possible mechanism for the formation of oligo(amylene) from fusel oil and zinc dihalide salts.

Zinc dihalides are known to form complexes with alcohols which are acidic in nature and such a complex would likely form between fusel alcohols and zinc bromide.^37^ The presence of isoamylene in the crude product mixture provides evidence in support of a possible mechanism for the formation of oligo(amylene) involving dehydration/polymerization of isoamyl alcohol, Fig. 9. Other researchers have shown that zinc chloride can bring about dehydration of sorbitol into isosorbide among other products.^38^ The isoamylene could then react with the acidic Lewis acid complex (H-complex) to form cationic intermediates which would bring about polymerization.^39–41^ The uncomplexed zinc dihalide could also polymerize the isoamylene since Antsus and Petrov made oligo(propene) by heating propene with zinc chloride under pressure.^42^

After removing the crude product from the reaction mixture, the zinc bromide was only contaminated by a small quantity of higher boiling oligo(amylene). The impure zinc bromide was effective in transforming a second portion of fusel oil into oligo(amylene) although the yield decreased slightly. The zinc bromide was readily purified from organic contamination by liquid phase extraction with water followed by azeotropic drying.^43^ We continue to study the recovery and recycling of zinc bromide for the dehydration/oligomerization of fusel oil and will report those details later. Since dehydration of the alcohol by zinc dihalide is believed to be the initial step of the process, the reaction conditions were altered to attempt the isolation of this product. Thus, anhydrous isoamyl alcohol was carefully added dropwise into stirred molten zinc chloride. However, the liquid that immediately distilled away was simply the unchanged alcohol.

### Preliminary fuel property analysis of the triamylene (oligo(amylene) *n* = 1)

Since the triamylene (oligo(amylene) *n* = 1) was readily isolated by one distillative fractionation of the oligo(amylene) mixture, the preliminary fuel property testing was carried out on this oligo(amylene) fraction. The boiling range, flashpoint, net heating value, cetane number, viscosity, density and freezing point of the triamylene were compared with those specifications for gasoline, diesel (D#2) and jet fuel (A-1), Table 2.^44–46^ The general ranges of carbon atoms for these reference fuels show that triamylene (∼C_15_) falls towards the kerosene range (D#2 and A-1).^47^ Although, it must be mentioned that gasoline, diesel and jet fuel are mixtures not only of alkanes but may contain up to 50% of cycloalkanes and aromatic hydrocarbons. Even when cooled to −78 °C, the triamylene remained a clear, mobile liquid when shaken and could be poured or even pipetted with a standard laboratory Pasteur pipette. Thus the triamylene had a low freezing point well below the specifications of the standard petroleum-based fuels, particularly aviation and diesel.^48,49^ The flash point of triamylene was 38 °C which is also the minimum for A-1 but somewhat less than D#2 (55 °C) and much higher than gasoline (−43 °C). The atmospheric pressure distillation of triamylene was over a range of 50–135 °C which was similar to the range for gasoline (50–200 °C) but lower than the boiling ranges of D#2 and A-1. The net calorific value of the triamylene fraction (43.96 kJ g^−1^) had ∼3% greater energy than the specifications of the three petroleum reference fuels (42.8 kJ g^−1^). The cetane number of the triamylene (31.9) did not meet the cetane number criterion for D#2 (40) or the related kerosene A-1 (48).^49^ However, the cetane number of the triamylene was almost double that of the ideal gasoline hydrocarbon 2,2,4-trimethylpentane (17.6).^50^ Although the precise molecular structure of the triamylene was not determined in this study, the hydrocarbon is undoubtedly highly branched given the proposed mechanism of formation. Thus, it was not surprising that the cetane number of the triamylene was only 31.9 since branching in alkanes increases ignition delay under compression ignition conditions (*e.g.* knocking). For example, the highly branched alkane 2,3,4,5,6,7,8-heptamethylnonane (C_16_) has a cetane number of 15.^51^ The viscosity (40 °C) of the triamylene (1.75 mm^2^ s^−1^) was slightly below the specifications for D#2 (1.9–4.1 mm^2^ s^−1^). The triamylene (2.53 mm^2^ s^−1^) was more viscous than gasoline (0.72 mm^2^ s^−1^) when measured at 20 °C.^52^ However, the viscosity of the triamylene (7.85 mm^2^ s^−1^) was approximately equivalent to the specifications of A-1 (8 mm^2^ s^−1^) at −20 °C. The density of the triamylene (0.78 g mL^−1^) was less than the specification for D#2 (0.85 g mL^−1^) at 15 °C but was within the range limits for both A-1 and gasoline.^53^ The triamylene (43.94 kJ g^−1^) had 8.5% more energy and nearly double the cetane number of isoamyl alcohol (37 kJ g^−1^ and 18.4, respectively), the major alcohol found in fusel oil from which it was made.^54,55^

**Table 2:** Selected fuel properties of triamylene compared to specifications of gasoline, diesel #2 and jet A-1

Fuel Property		Gasoline^a^	Diesel #2^b^	Jet A-1^c^	Triamylene
Carbon atoms		5–13	10–25	9–13	14–16
Boiling range (°C)		50–200	200–300	140–280	50–135
Flashpoint (°C)		−43	≥55	≥38	38
Net heating value (kJ g^−1^)		42.8	42.7	42.8	43.94
Cetane number		17.6^d^	≥40	48^e^	31.9
Viscosity (mm^2^ s^−1^) (°C)	40	—	1.9–4.1	—	1.75
	20	0.72^f^	—	—	2.53
	−20	—	—	≤8	7.85
Density (g mL^−1^, 15 °C)		0.71–0.78^g^	0.85	0.78–0.84	0.78
Freezing point (°C)		−60^h^	−12^i^, −40^j^	≥−47	<−78

a Ref. 44.

b Ref. 45.

c Ref. 46.

d 2,2,4-Trimethylpentane (ref. 50).

e Ref. 49.

f Ref. 52.

g Ref. 53.

h Ref. 48.

i Cloud point (ref. 49).

j Pour point (ref. 49).

## 4. Conclusions

Heating mixtures of fusel oil with zinc chloride or zinc bromide does produce the deoxygenated hydrocarbon oligo(amylene) as reported by Balard though the yield was only ∼25%. The major product from the reaction was diisoamyl ether from etherification of isoamyl alcohol, the major component of fusel oil.^56^ The reaction procedure was uncomplicated and although zinc chloride or zinc bromide were employed stoichiometrically, the salts could be recovered and recycled quantitatively. Unlike noble metal catalyst employed in several ATJ processes, the zinc salts are inexpensive since they are derived from a ‘coinage’ metal.^57^ The zinc salts were robust and unaffected by the presence of water in the fusel oil. Although the ether co-product may be useful as a diesel oxygenate, it was not desirable for our purposes.^7^ The process was demonstrated at modest scale to prepare oligo(amylene) (100 mL) but the challenges of isolating the hydrocarbon will require further study and optimization. The triamylene fraction of the oligo(amylene) (*n* = 1) is less useful for compression ignition engines since the cetane number was lower than specifications for US diesel #2. The triamylene had net calorific value slightly better than the minimum specifications for diesel #2, jet A-1 and gasoline. Other fuel property data for the triamylene such as flash point, boiling range, viscosity, freezing point and density compared reasonably well to those of jet A-1. Thus, the triamylene could be a fungible blendstock for gas turbine engine fuels.^58,59^ By controlling the degree of oligomerization, the fusel oil can be converted into fuels or lubricants. The heavier oligomers which are less desirable as transportation fuels could be useful base oils for lubricant fluids, greases and hydraulic fluids. The oligo(amylene) could be used in crude form for burning in less discriminating engines such as boiler units and generators that operate on Class C2 fuel oil.^60^ The oligo(amylene) described here was made in batches but experiments are currently underway to run the process continuously for increased throughput.

## Conflicts of interest

There are no conflicts to declare.

## Supplementary Material

RA-011-D0RA10386A-s001
