# Supplementary material for: Oligo(amylene) from the reaction of fusel oil with zinc dihalide
Source: RSC Adv. 2021 Jan 7;11(4):1960–8. doi: 10.1039/d0ra10386a (PMC9134217; doi:10.1039/d0ra10386a)
Supplement: RA-011-D0RA10386A-s001 [file RA-011-D0RA10386A-s001.pdf]

Electronic Supplementary Material (ESI) for RSC Advances.
This journal is © The Royal Society of Chemistry 2020

ELECTRONIC SUPPLEMENTARY INFORMATION (ESI)

Oligo(amylene) from the Reaction of Fusel Oil with Zinc Dihalide

Matthew C. Davis[1], Laszlo Prokai[2], Josanne Dee Woodroffe[1]

[1]Naval Air Warfare Center, Michelson Laboratory, Chemistry Division, China Lake, California 93555, USA

[2]University of North Texas Health Sciences Center, Department of Pharmacology and Neuroscience, Mass Spectrometry and Proteomics Laboratory, 3500 Camp Bowie Boulevard, Fort Worth, Texas 76107, USA

Gas chromatography–mass spectrometry (GC-MS)

Analyses were conducted on a TRACE 1300 gas chromatograph equipped with an AI 1310 autoinjector and interfaced to an ISQ 7000 quadrupole mass spectrometer (Thermo Scientific, San Jose, CA). A 30 m × 0.25 mm i.d. column with 0.25 µm thick TG-5SILMS stationary phase (5% phenylsiloxane-containing methylsiloxane; TraceGold™, Thermo Scientific) was used for the separation of the reaction products at 1.5 mL/min flow rate of the helium carrier gas. Column oven temperature was isothermal at 40 °C for 3 min, then raised to 350 °C at 3 or 10 °C/min rate and held at 350 °C for 20 min. Samples (0.1 µL aliquots of neat liquids) were injected in split mode with 1:100 split ratio and injector temperature of 275 °C. The mass spectrometer was operated with electron ionization (EI), and electron energy set to either 70 eV (standard) or 12 eV (to reduce EI-associated fragmentation of molecular ions). Ion source and transfer line temperatures were 250 °C and 300 °C, respectively. Mass spectra were acquired in the *m/z* 35–500 range with cycle time of 0.2 s. The system was controlled by Thermo Scientific's XCalibur software (version 4.0), and data were processed with the Qual Browser module of the program.

Bomb Calorimetry

The gross heat of combustion (H_c), or calorific value, of the oligo(amylene) (n = 1) was obtained using a Parr 6725 Semi-micro Calorimeter outfitted with a Parr Calorimeter Thermometer according to the standard method.[1] Benzoic acid was used as the internal standard (e.g., spike) for the tests and to calculate the energy equivalent of the instrument. Between each combustion measurement, the oxygen bomb was disassembled and cleaned with distilled water. The gross heat of combustion (H_c) was calculated from the average of three separate measurements. From the H_c (kJ/g), the net heat of combustion (H_n) in kJ/g was calculated Eq. 1:[2,3]

$$H_n = H_c - [21.96 \text{ x (weight fraction hydrogen of the analyte)}] \qquad (1)$$

This equation deducts the enthalpy of vaporization of water, resulting from the complete oxidation of the hydrogen contained in the analyte.

Ignition Quality Test (IQT) and Derived Cetane Number (DCN)

The derived cetane number of the oligo(amylene) (n = 1) (sample volume 100 mL) was collected by the Southwest Research Institute® (San Antonio, TX) using an Ignition Quality Test apparatus according to the published method.[4] The ignition delay time (τ) for the fuel was measured (ms) and averaged over 32 combustion cycles. The DCN value was then calculated based on τ and the equations found in the standard method.

Elemental Microanalysis

In further support for the chemical identity and purity of the oligo(amylene) made in this report, elemental microanalysis (C and H) of the product was made by Atlantic Microlab, Inc. (Norcross, GA).

Freezing Point

Analytical instrumentation was unavailable for measurement of the melting point of the oligo(amylene) (n = 1). Therefore, the oligo(amylene) (n = 1) (5 mL) was placed in a glass vial which was incubated in a dry ice/acetone bath (–78 °C) for 1 h as a qualitative test of phase change.

Flash Point

The flash point data for the oligo(amylene) (n = 1) (sample volume 2 mL) was collected with a Grabner Instruments MiniFlash FLP by the closed cup method.[5]

Density and Kinematic Viscosity

Density and kinematic viscosity of the oligo(amylene) (n = 1) was obtained simultaneously using an Anton-Paar SVM 3001 Stabinger viscometer by the published method.[6] The instrument was injected with analyte (~3 mL) and set to run from –20 to 40 °C in 5 °C increments.

Nuclear Magnetic Resonance Spectroscopy

Nuclear magnetic resonance (NMR) spectroscopy of the oligo(amylene) was performed to ensure the correct chemical constitution and qualitative purity. Solutions for NMR analysis were made by dissolving the analyte (~20 mg) in deuterated chloroform ($CDCl_3$, 1 mL). All data were collected on a JEOL ECZ 400 MHz spectrometer (^{1}H at 400 MHz, ^{13}C at 100 MHz). Nuclear magnetic resonance data (free-induction decay's) were processed using ACD/NMR Processor software (Advanced Chemistry Development Inc., Toronto). All spectra were referenced to solvent or tetramethylsilane.

References

1. ASTM D240-17 (2017) *Standard test method for heat of combustion of liquid hydrocarbon fuels by bomb calorimeter.* ASTM International, West Conshohocken, Pennsylvania, USA.
2. V. Babrauskas, Related quantities (a) heat of combustion and potential heat. In: V. Babrauskas and S. J. Grayson (eds) *Heat release in fires*, Elsevier Applied Science, London, 1992.
3. R. S. Jessup, Precise measurement of heat of combustion with a bomb calorimeter. U.S. Department of Commerce, National Bureau of Standards, Washington, 1960. http://www.dtic.mil/dtic/tr/fulltext/u2/a286701.pdf. [accessed 28 Aug 2018]
4. ASTM D6890-16e2 (2018) *Standard test method for determination of ignition delay and derived cetane number (DCN) of diesel fuel oils by combustion in a constant chamber*. ASTM International, West Conshohocken, Pennsylvania, USA.
5. ASTM D7094-17a (2017) *Standard test method for flash point by modified continuous closed cup (MCCCFP) tester*. ASTM International, West Conshohocken, Pennsylvania, USA.
6. ASTM D4052-18 (2018) *Standard test method for density, relative density, and API gravity of liquids by digital density meter*. ASTM International, West Conshohocken, Pennsylvania, USA.

Table 1 Bomb calorimetry data on the oligo(amylene) (n = 1).

Run	Mass (g)	Gross HoC[1] (kJ/g)	Net HoC (kJ/mol)	Volumetric Net HoC (MJ/L)	Average (MJ/L)	Standard deviation	RSD <1%	Gravimetric Net HoC (MJ/kg)
1	0.3171	47.0419	4410.820	34.417				
2	0.2710	46.7779	4384.420	34.211	34.290	0.111	0.323	43.946
3	0.2820	46.82	4388.630	34.244				

[1] HoC = heat of combustion

Table 2 Viscosity and density of the oligo(amylene) (n = 1)

Cell T(°C)	Kinematic Viscosity (mm^2/sec)	Density (g/mL)
40	1.75	0.762
35	1.90	0.766
30	2.08	0.769
25	2.28	0.773
20	2.53	0.777
15	2.81	0.780
10	3.15	0.784
5	3.56	0.788
0	4.06	0.791
–5	4.71	0.794
–10	5.50	0.798
–15	6.51	0.802
–20	7.85	0.805

Table 3 Ignition quality test (IQT) data and derived cetane number (DCN) for the oligo(amylene) (n = 1)

Avg. Air Temp. (°C)	Ignition Delay (ms)	DCN
555.8	6.71	31.9

EI-MS (70 eV) of methylbutane peak

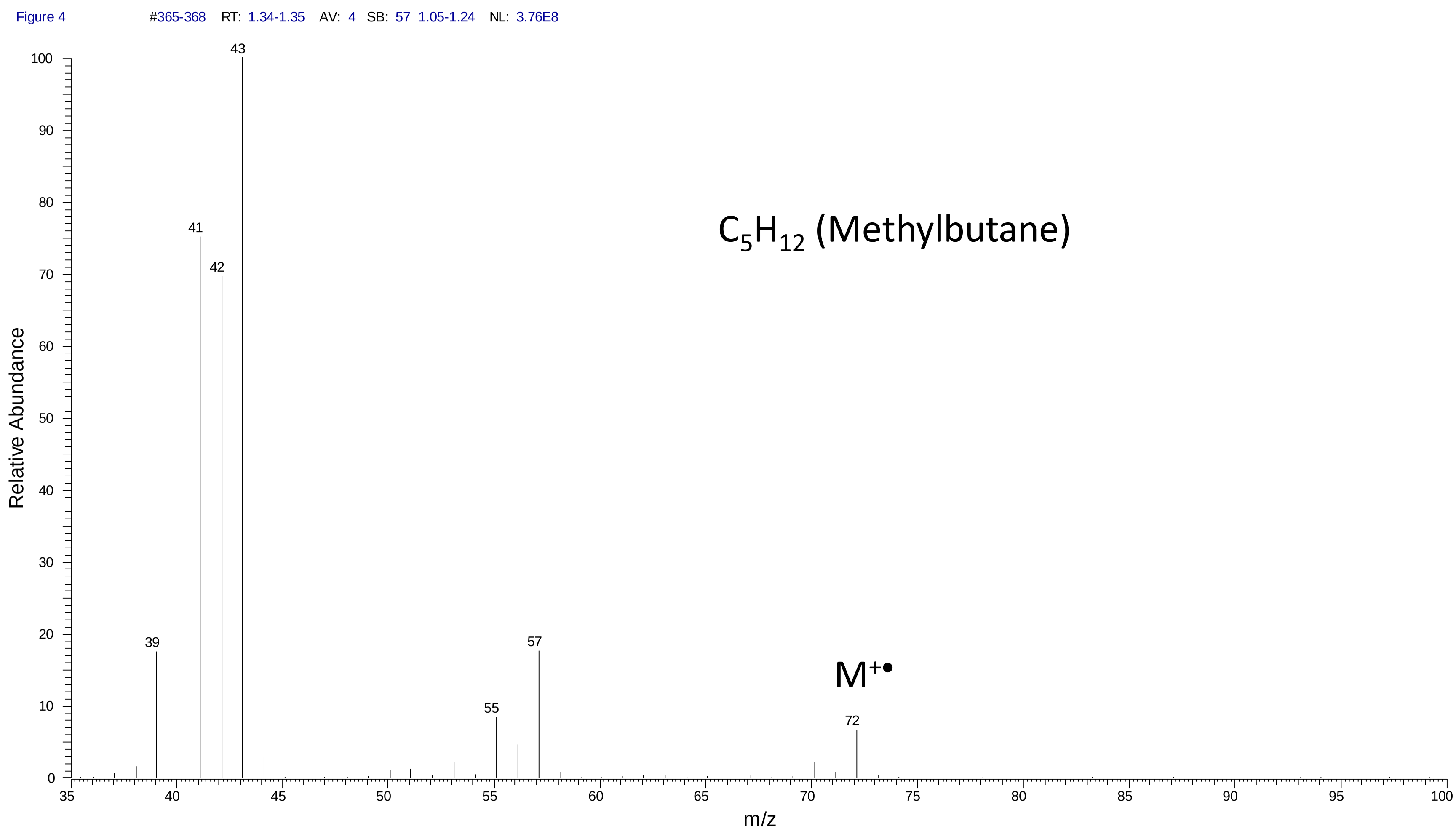

EI-MS (70 eV) of methylbutene peak

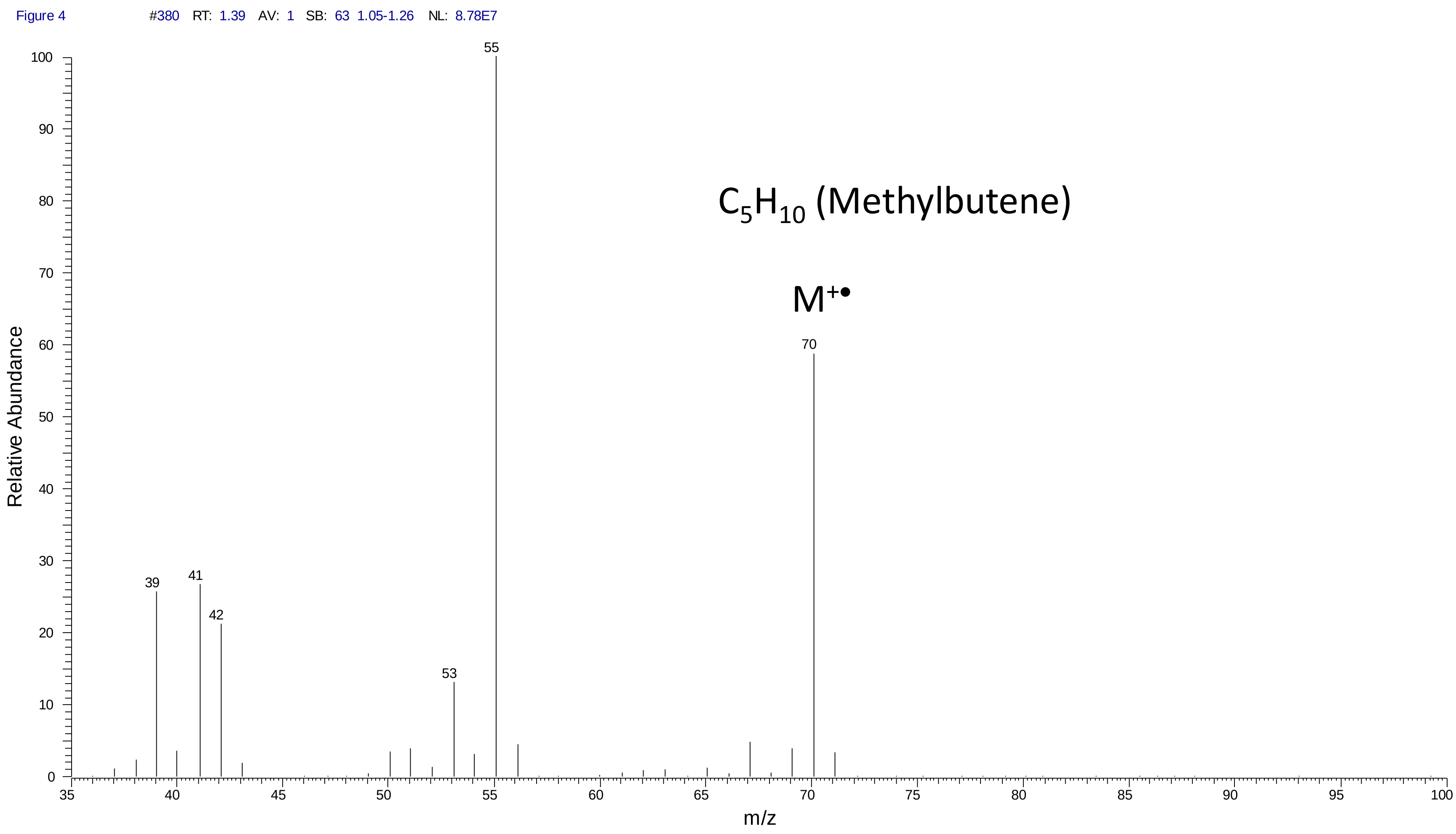

EI-MS (70 eV) of isoamyl alcohol peak

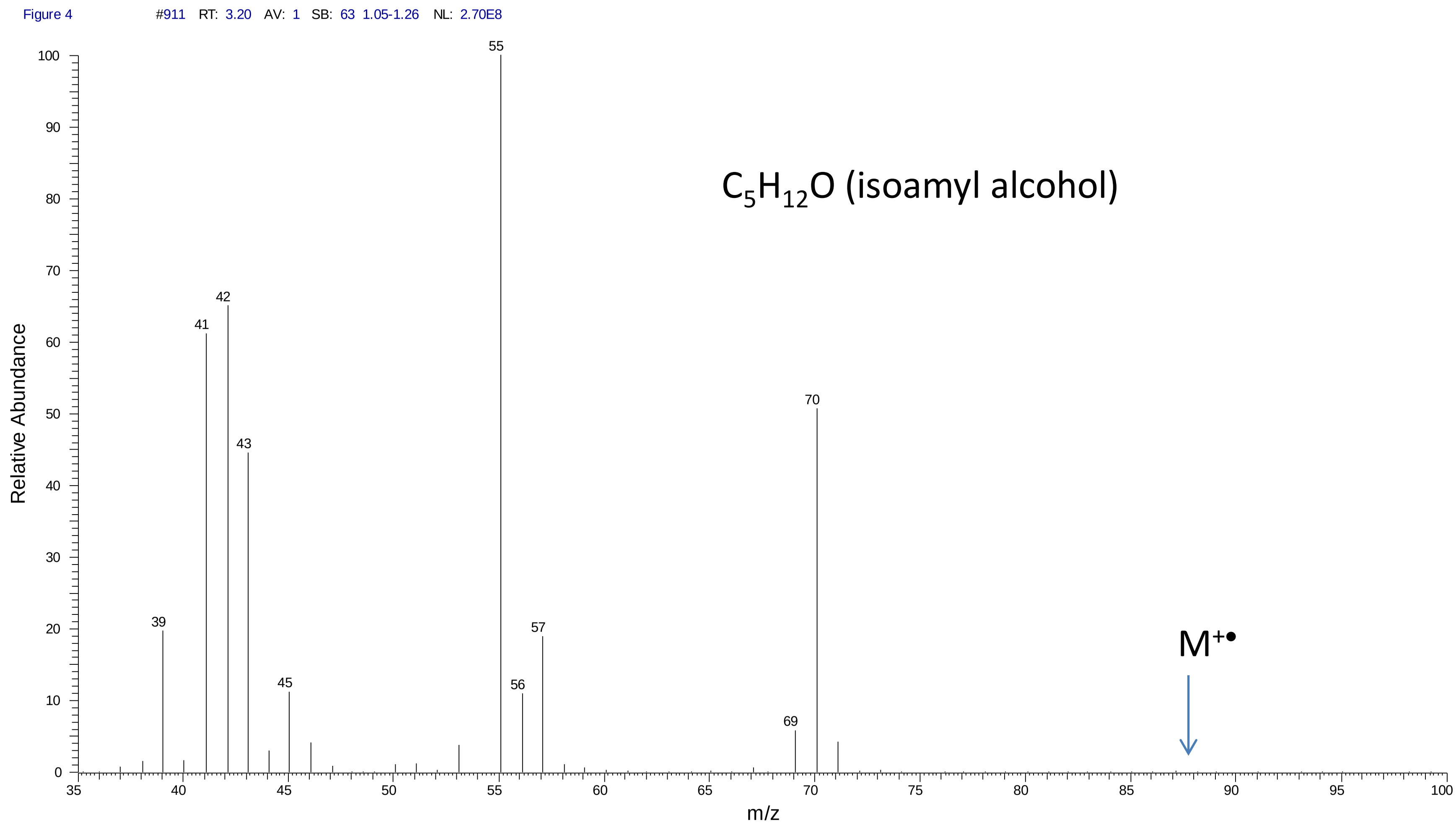

EI-MS (70 eV) of isoamyl bromide peak

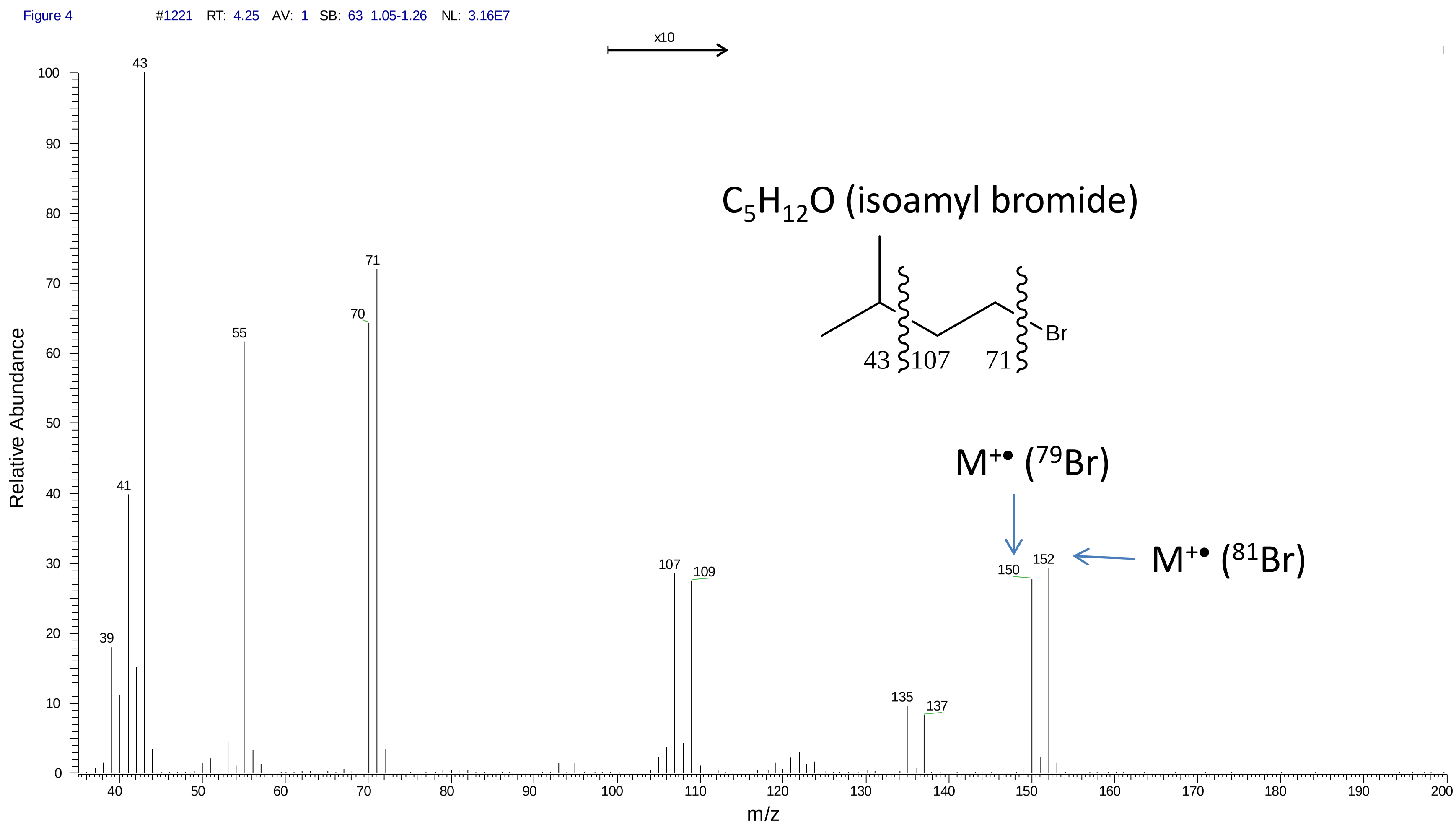

EI-MS (70 eV) of diisoamyl ether peak

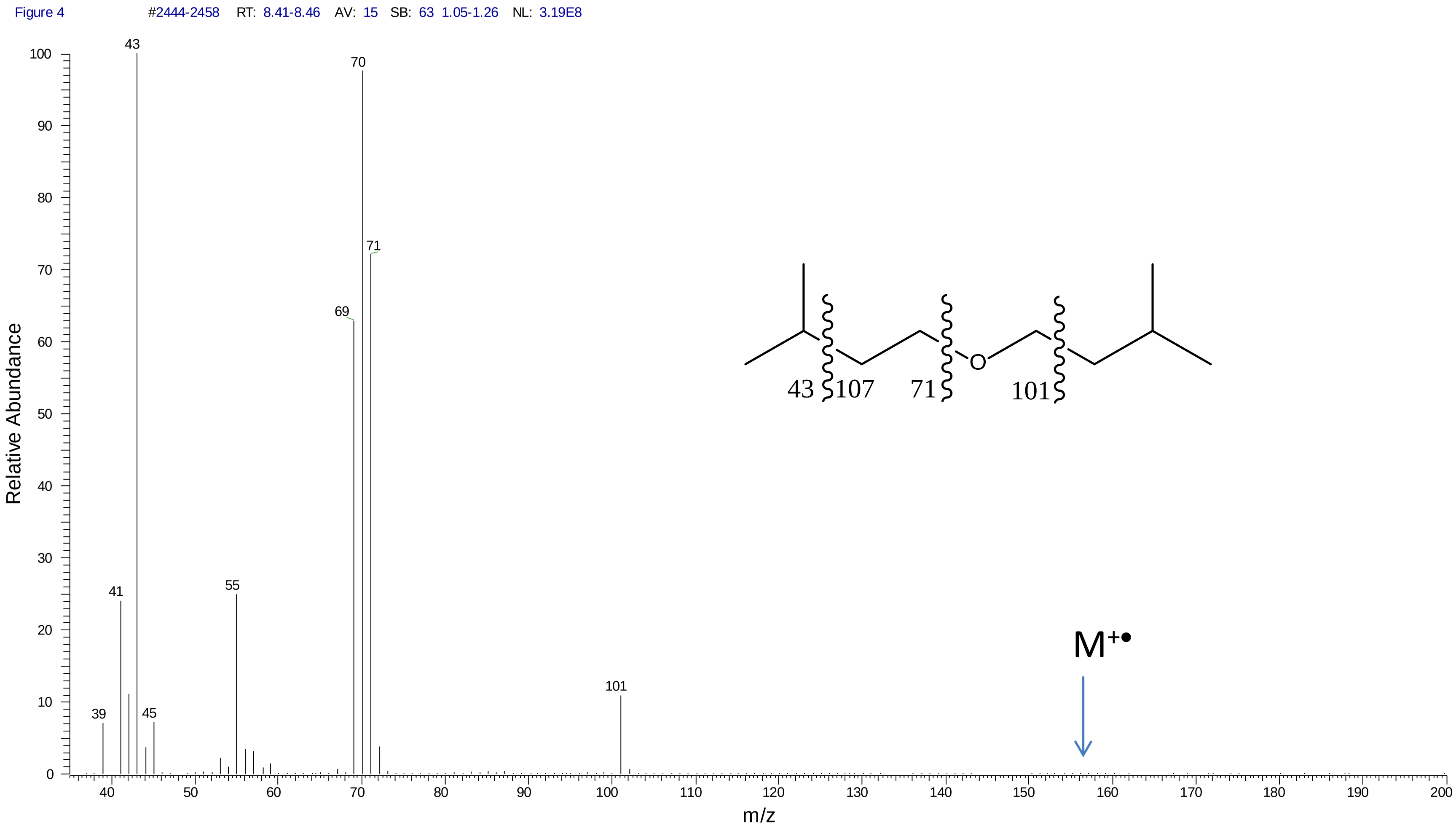

EI-MS (70 eV) of peak from the diamylene (n=0) domain (RT: 5.74 min)

Figure 4 #1659 RT: 5.74 AV: 1 SB: 63 1.05-1.26 NL: 1.45E7

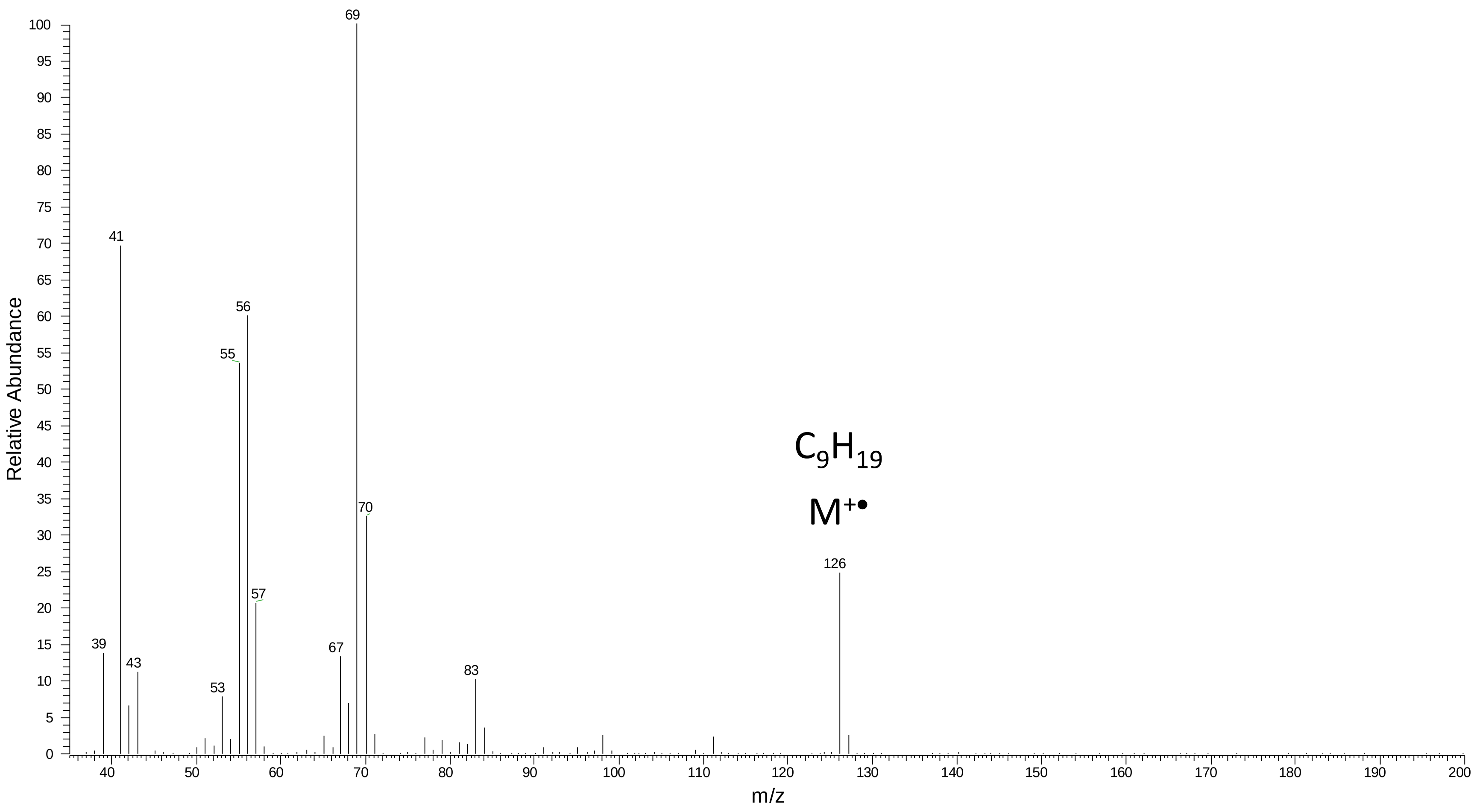

EI-MS (70 eV) of peak from the diamylene (n=0) domain (RT: 7.58 min)

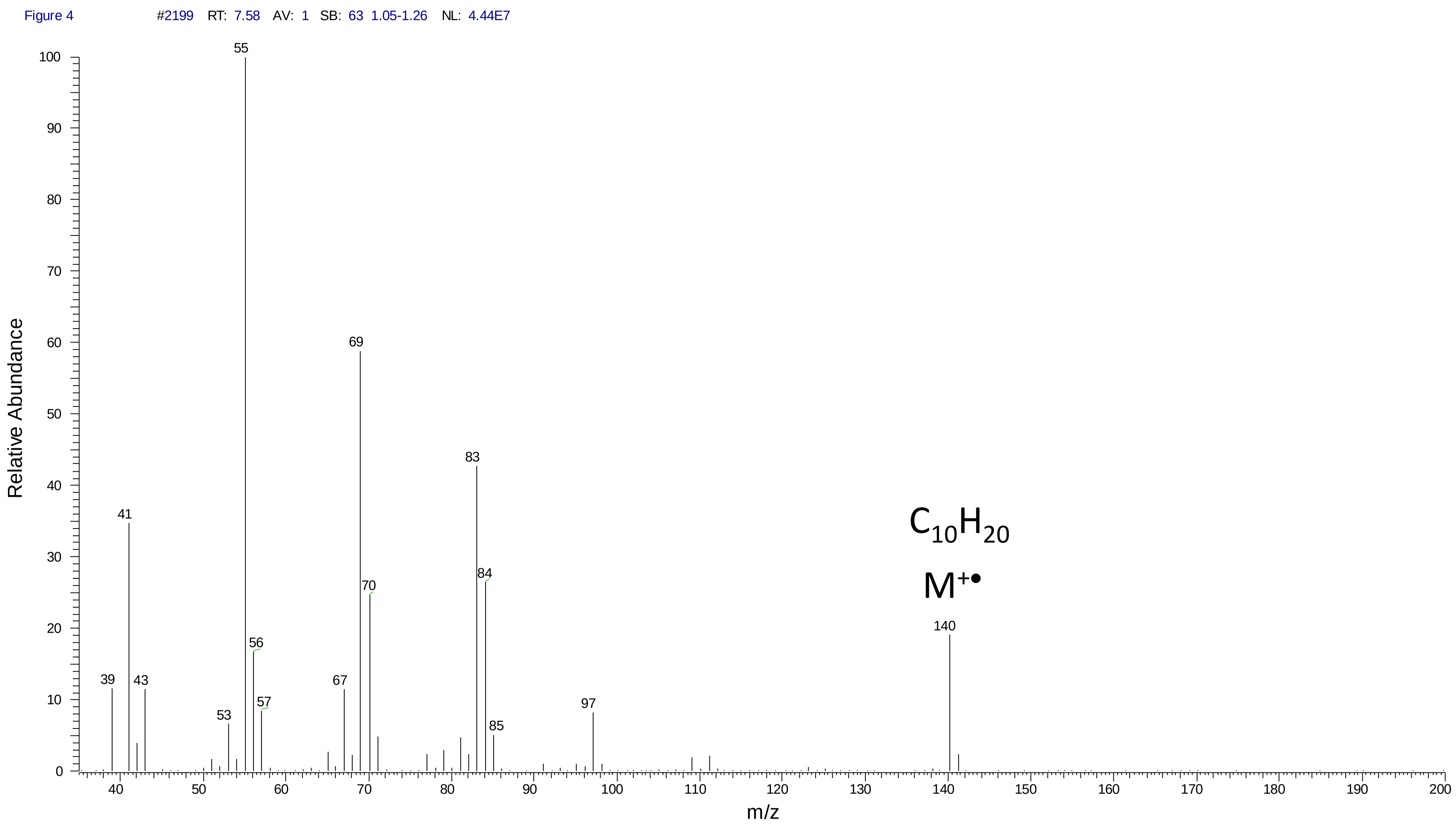

EI-MS (70 eV) of peak from the diamylene (n=0) domain (RT: 9.14 min)

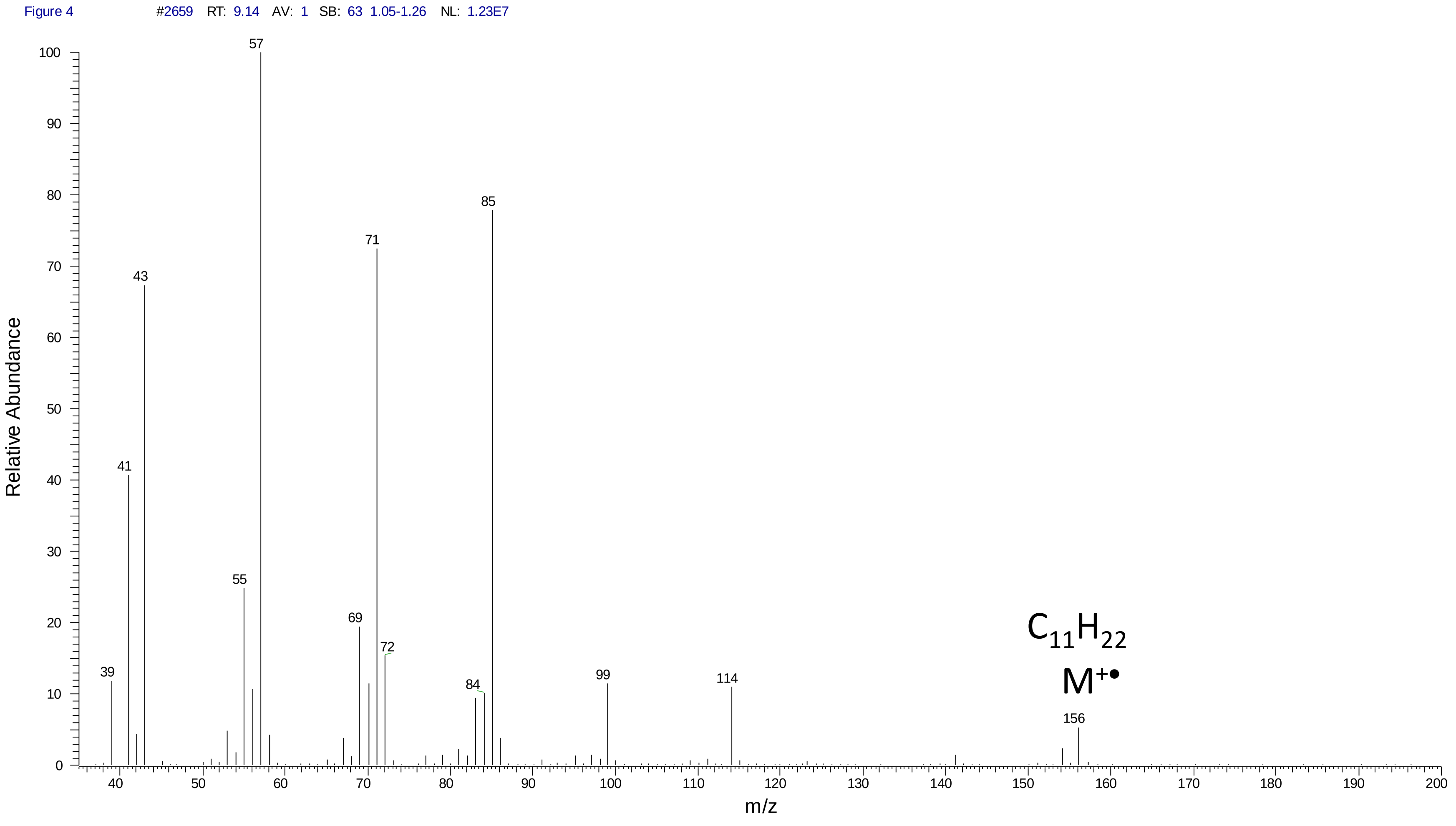

Averaged EI-MS (12 eV) of the diamylene (n=0) domain

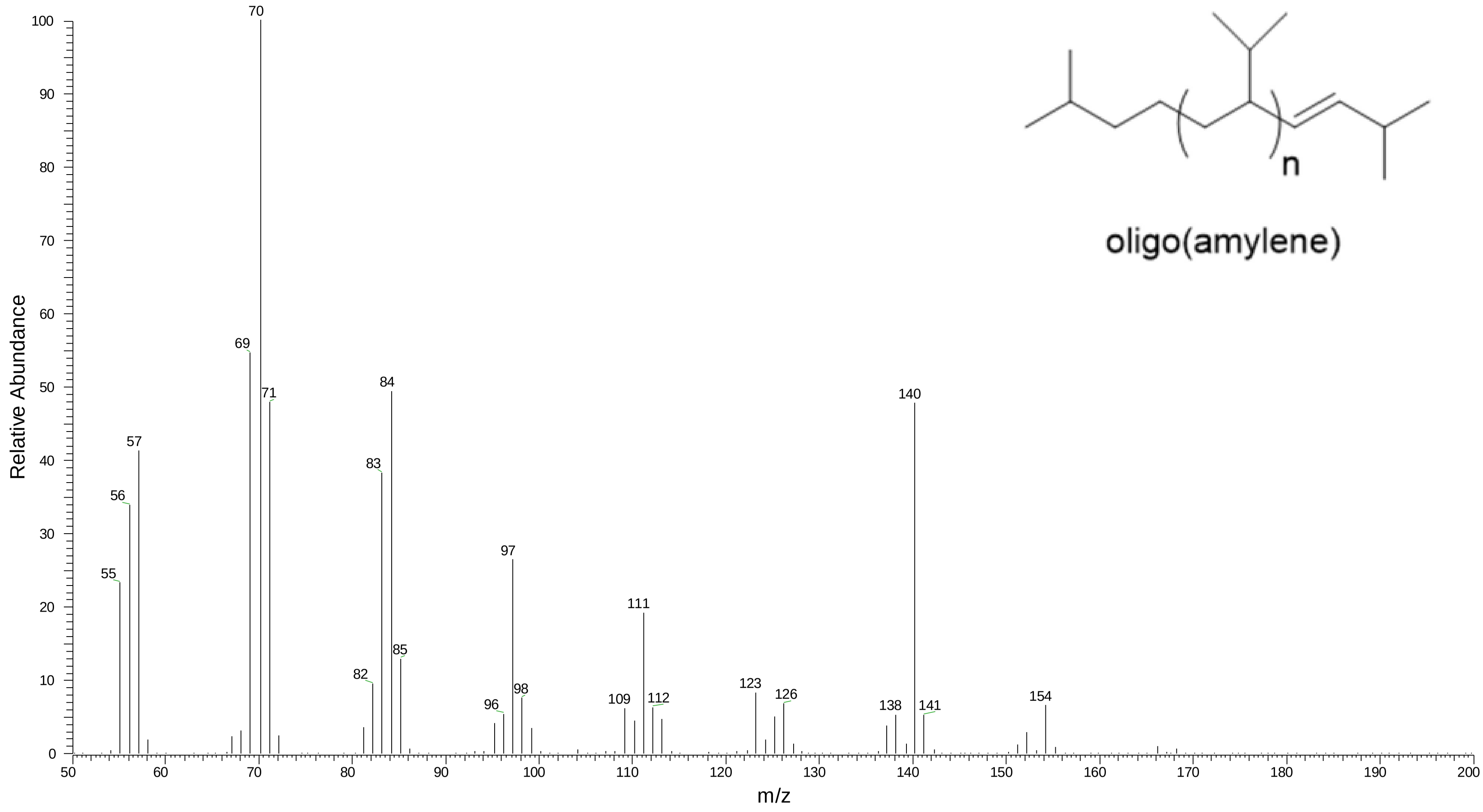

oligo(amylene)

Averaged EI-MS (12 eV) of the tetraamylene (n=2) domain

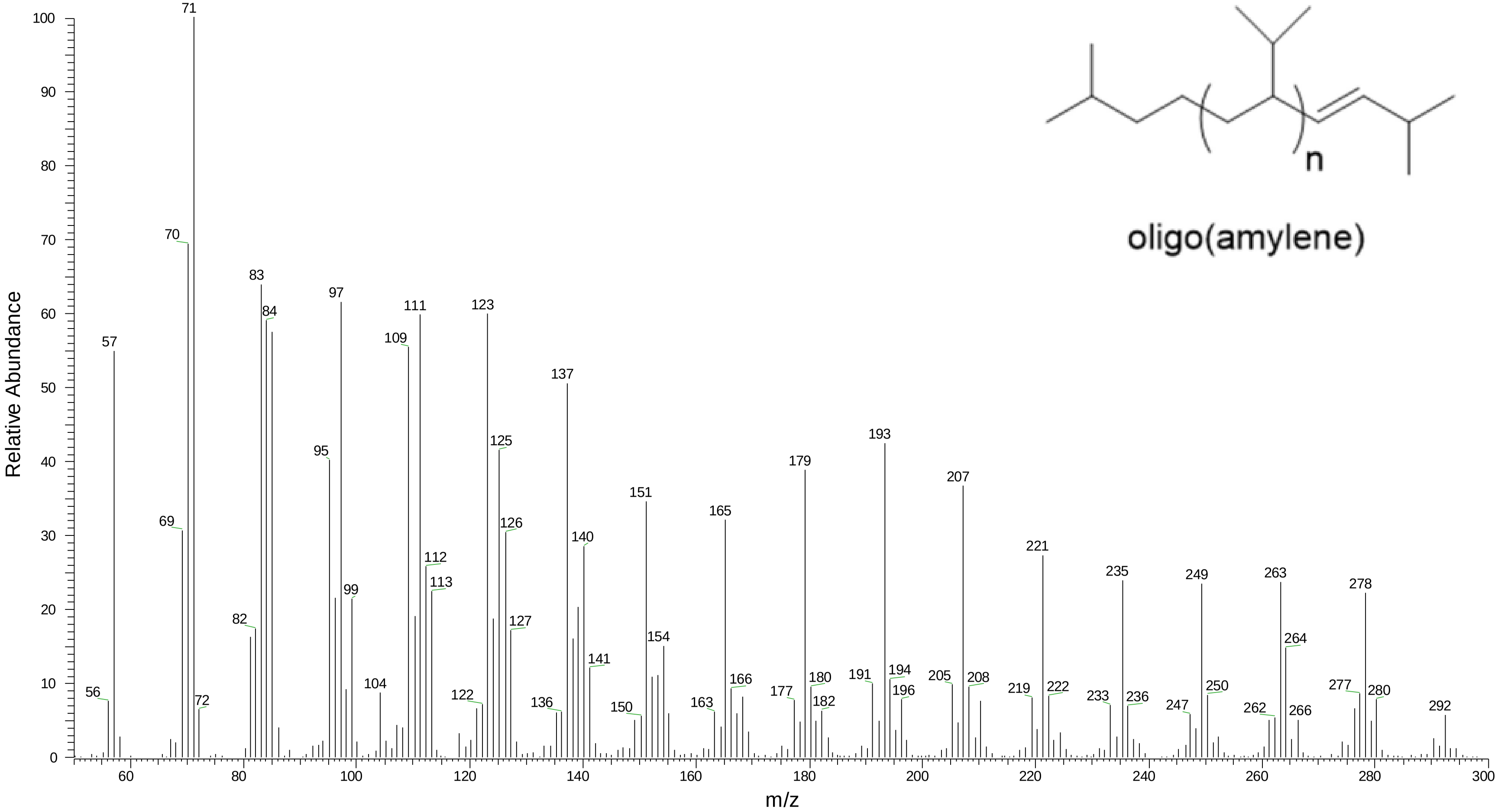

oligo(amylene)

Averaged EI-MS (12 eV) of the pentaamylene (n=2) domain

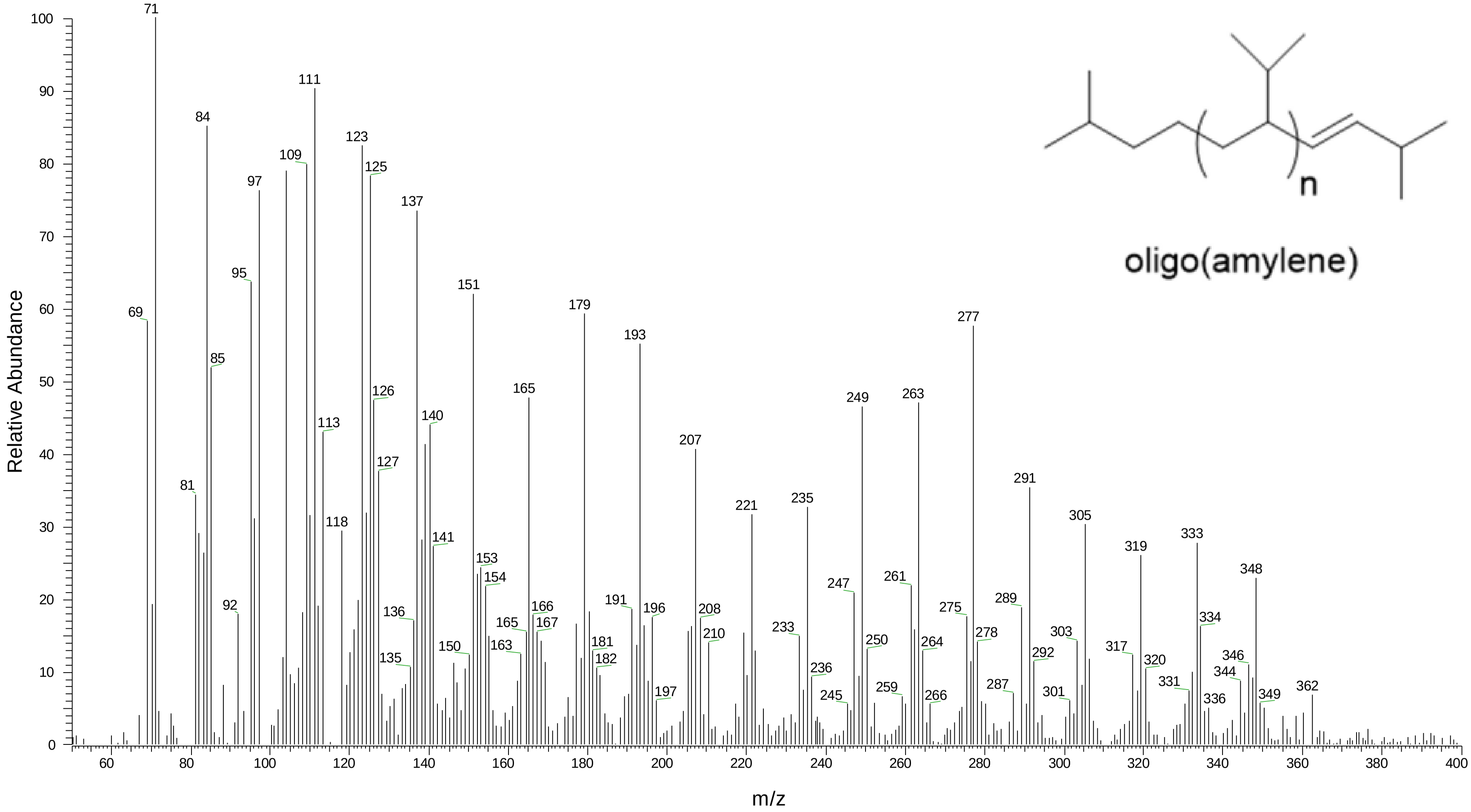

oligo(amylene)

Total-ion chromatogram (TIC) pot residue oligo(amylene) fractionation

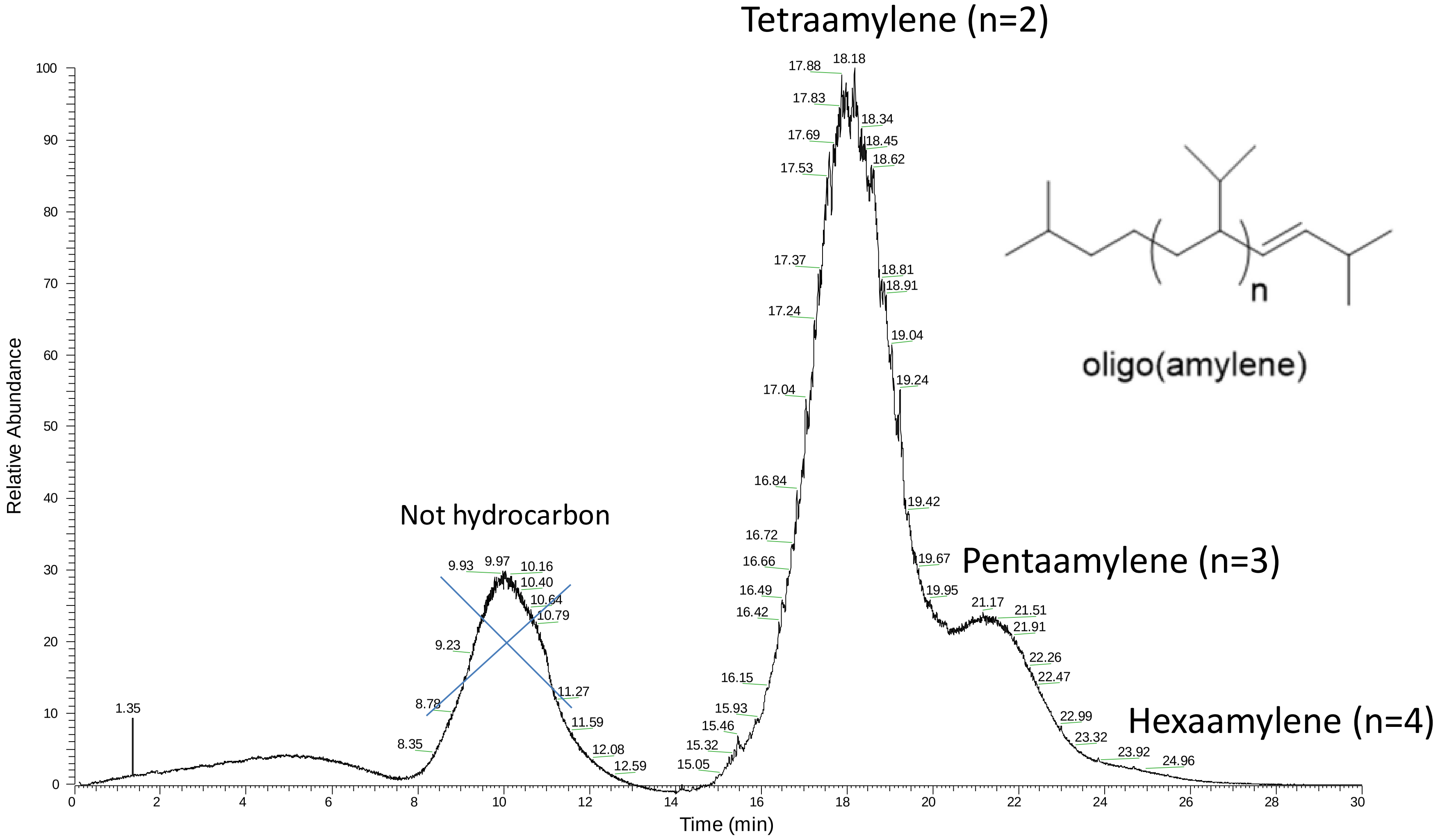

Averaged EI-MS (12 eV) of the tetraamylene (n=2) of pot residue

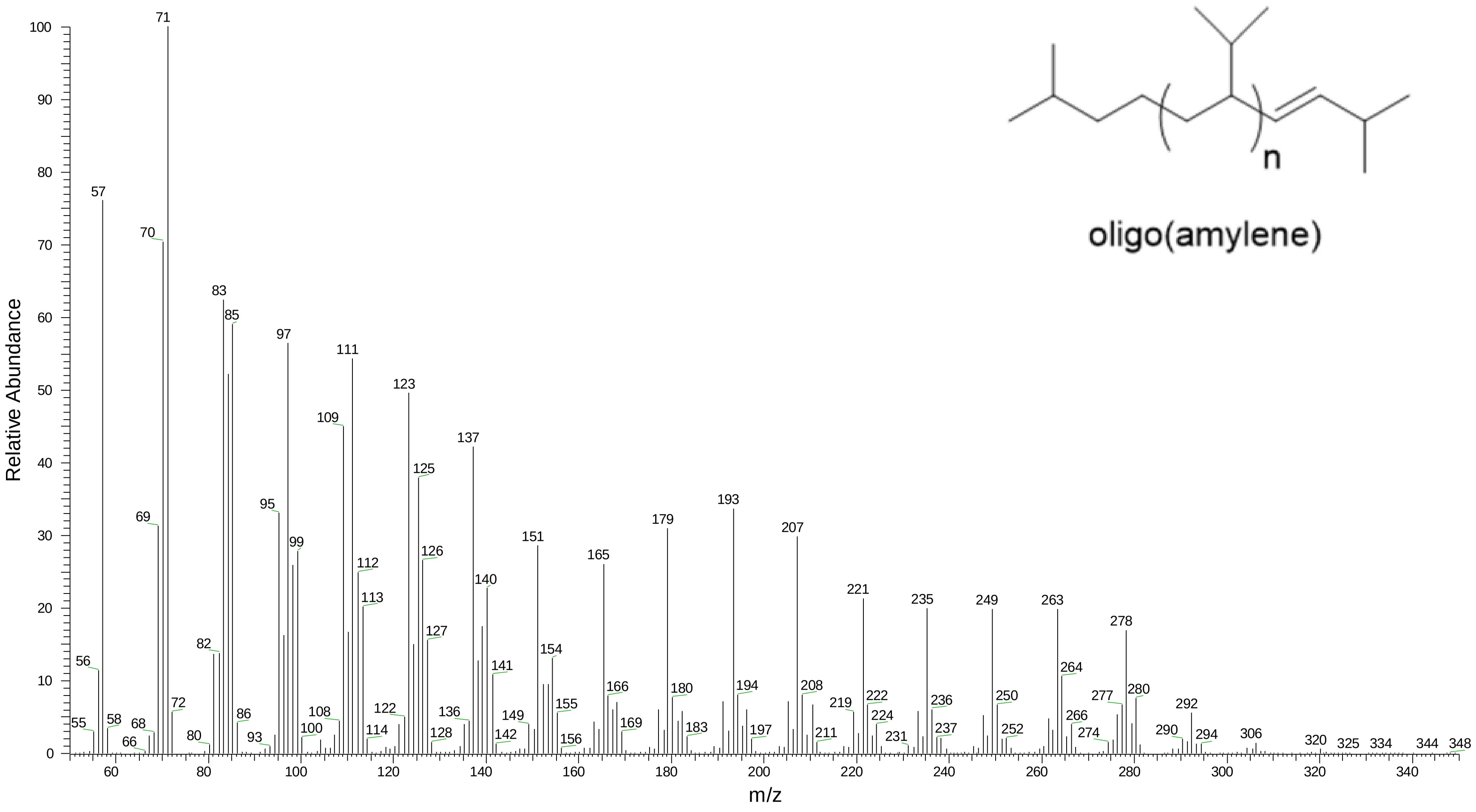

oligo(amylene)

Averaged EI-MS (12 eV) of the pentaamylene (n=3) of pot residue

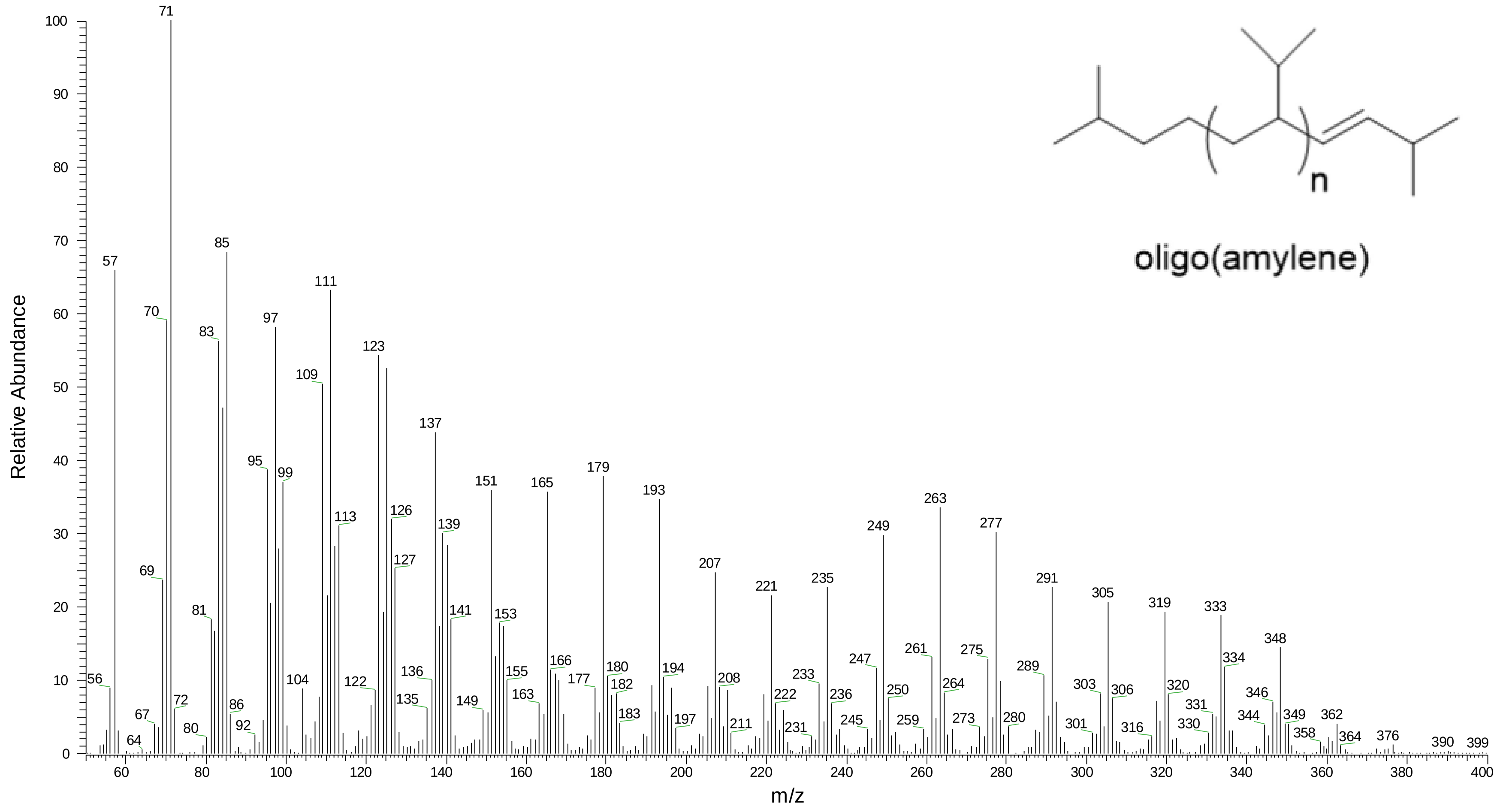

oligo(amylene)

Averaged EI-MS (12 eV) of the triamylene (n=1) in Figure 8

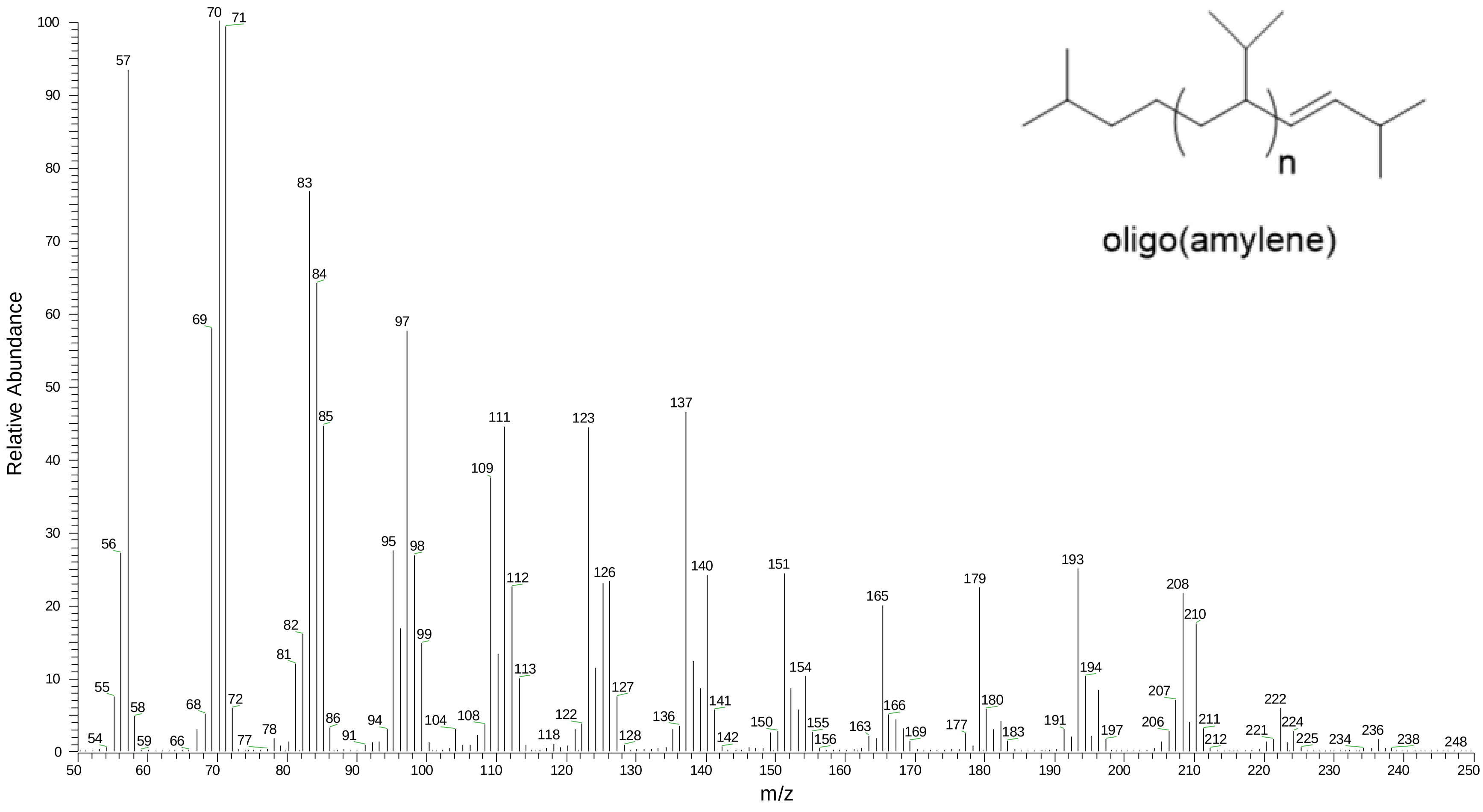

Total-ion chromatogram (TIC, 70 eV) of tetraamylene from overnight heat

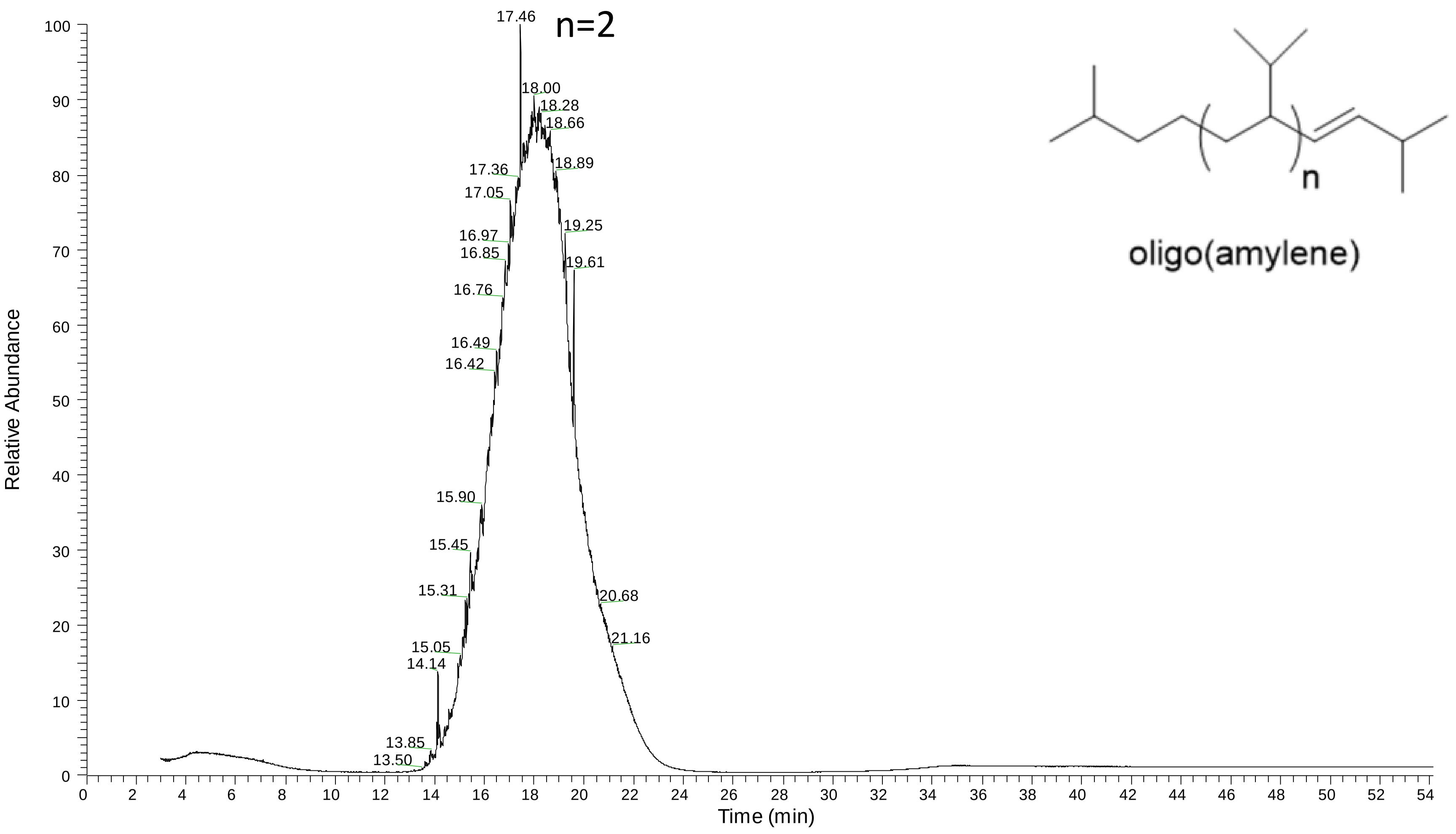

Averaged EI-MS (12 eV) of tetraamylene from overnight heat

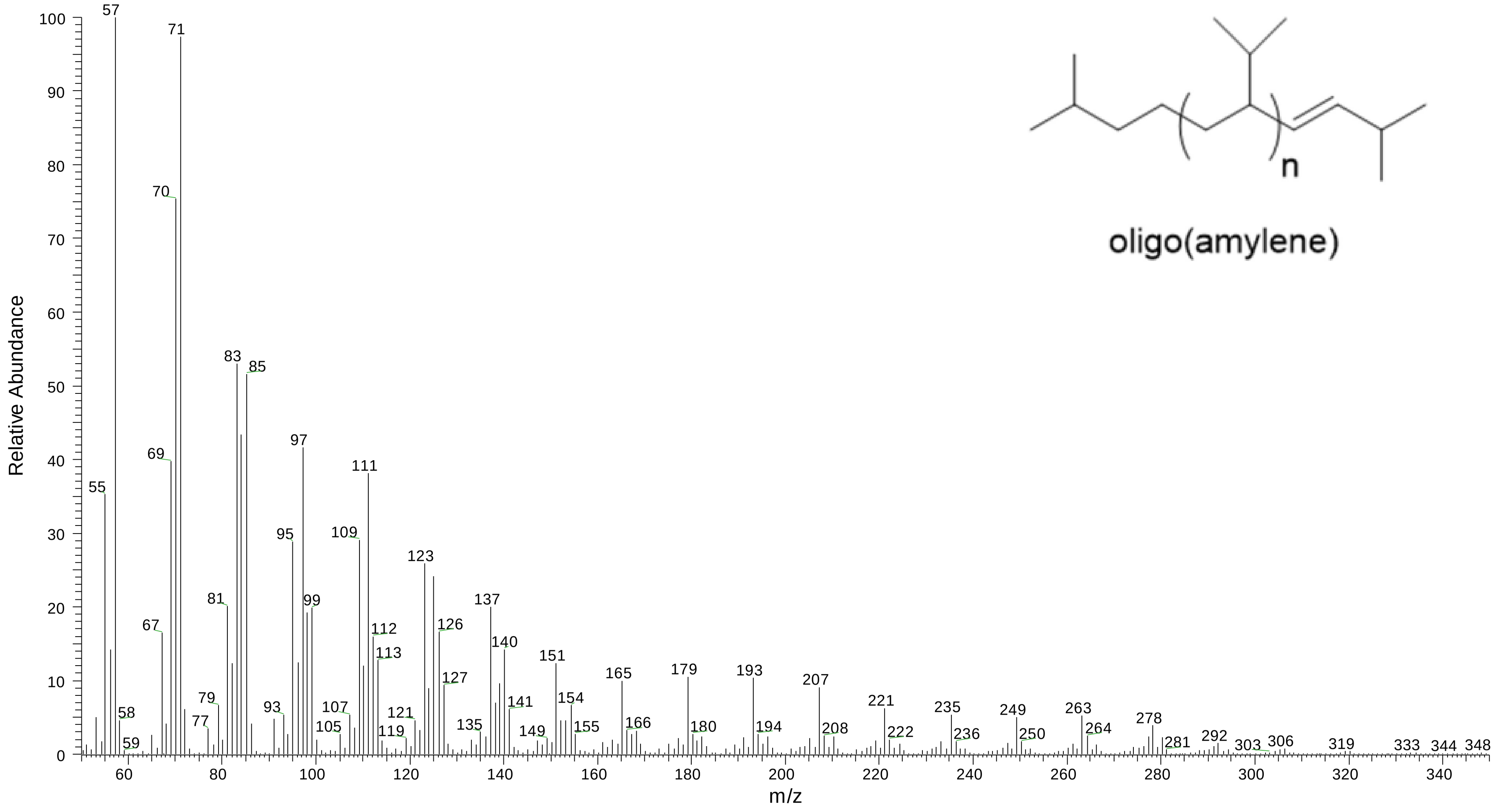

oligo(amylene)
